# Crush Syndrome and Systemic Necrosis in Trauma Patients: A Systematic Review of Pathophysiology, Anatomical Impact, and Renal Outcomes

**DOI:** 10.7759/cureus.89592

**Published:** 2025-08-07

**Authors:** Hagwa Gafar Abubakir Osman, Mohammed Elfatih Elbadri, Ibrahim Adil Hamadelniel Alhadi, Ahmed S Ibrahim, Aisha Ayyub, Ahmed Mohamed, Abdelrahman Sahnon Abaker Sahnon, Jarallah H. J. Alkhazendar, Aliaa H Alkhazendar, Manahil Awan

**Affiliations:** 1 General Practice, Badr Al Samaa Hospital, Makkah, SAU; 2 Orthopedics, Khorfakkan Hospital, Sharjah, SAU; 3 General Surgery, Royal College of Surgeons of Edinburgh, Edinburgh, GBR; 4 Human Anatomy, University of Gezira, Wad Madani, SDN; 5 General Surgery, University of Gezira, Wad Madani, SDN; 6 Orthopedics, Nile University, Khartoum, SDN; 7 Pathology, Sir Syed College of Medical Sciences for Girls, Karachi, PAK; 8 Orthopedics and Trauma, Gezira Center for Orthopaedic Surgery and Traumatology, Wad Madani, SDN; 9 General Practice, Medical Council of Ireland, Dublin, IRL; 10 General and Emergency Surgery, Lister Hospital, East and North Hertfordshire NHS Trust, Stevenage, GBR; 11 Surgery, Islamic University of Gaza, Gaza, PSE; 12 Surgery, Liaquat National Hospital, Karachi, PAK

**Keywords:** acute kidney injury, crush syndrome, ferroptosis, fluid resuscitation, hyperbaric oxygen therapy, ischemia-reperfusion injury, rhabdomyolysis, trauma

## Abstract

Crush syndrome remains a life-threatening complication of traumatic injuries, especially in mass casualty and disaster scenarios. This systematic review evaluates the current clinical and mechanistic understanding of crush-related pathophysiology, anatomical impact, and renal complications, with a focus on therapeutic interventions. Studies were selected using the PICO framework and analyzed under PRISMA guidelines. A total of six studies, including narrative reviews, clinical trials, and a systematic review, were included. Core findings highlighted ischemia-reperfusion injury, rhabdomyolysis, and ferroptosis as key drivers of systemic toxicity, often culminating in acute kidney injury (AKI). Anatomically, prolonged soft tissue compression and necrosis posed serious risks for long-term disability and systemic inflammation. Adjunctive therapies such as hyperbaric oxygen therapy (HBOT) demonstrated potential benefits in wound healing and tissue preservation, though evidence remains limited by study heterogeneity. Overall, this review offers an integrative synthesis of existing knowledge, identifies therapeutic gaps, and emphasizes the need for standardized, evidence-based protocols for managing crush syndrome.

## Introduction and background

Crush syndrome is a severe and life-threatening condition that develops after prolonged compression of skeletal muscle, commonly seen in trauma patients. It often occurs in situations such as earthquakes, vehicle collisions, or industrial accidents, where victims are trapped under heavy objects [1]. The sustained pressure causes muscle necrosis, leading to the release of toxic substances such as myoglobin, potassium, creatine kinase, and phosphorus into the bloodstream. This sudden release can trigger systemic complications including shock, hyperkalemia, disseminated intravascular coagulation (DIC), and acute kidney injury (AKI), which are major contributors to patient death and disability [2].

The underlying pathophysiology involves ischemia-reperfusion injury, where restricted blood flow to muscles causes hypoxia and cellular death, and restoration of circulation worsens the damage due to oxidative stress and inflammation [3]. These processes lead to metabolic acidosis, electrolyte imbalance, and myoglobin-induced nephrotoxicity. Among these, AKI is a critical concern, often requiring renal replacement therapy. Cardiac arrhythmias from hyperkalemia are also life-threatening. Treatment typically includes early and aggressive fluid resuscitation, correction of metabolic disturbances, and dialysis when necessary. Newer interventions such as hyperbaric oxygen therapy (HBOT), antioxidants, and agents that block ferroptosis are being explored for better outcomes [4].

Despite its severity, crush syndrome is often missed or diagnosed late, especially in mass casualty situations where rapid triage is challenging. Anatomical factors such as lower limb crush and compartment syndrome can worsen systemic effects. Management approaches differ widely, and there is no universal guideline for diagnosis or treatment. Although various studies have examined different aspects of the syndrome, such as tissue damage, renal injury, and therapeutic options, there is a need for a comprehensive review that combines all these aspects. This systematic review aims to bridge that gap by synthesizing current evidence on pathophysiology, anatomical damage, and renal complications in crush syndrome, helping guide future clinical and research efforts.

## Review

Materials and methods

Search Strategy

This systematic review adhered to the Preferred Reporting Items for Systematic Reviews and Meta-Analyses (PRISMA) 2020 guidelines [5] to ensure transparency and methodological robustness. A comprehensive literature search was conducted in the following four major biomedical databases: PubMed/MEDLINE, Embase, Scopus, and the Cochrane Library. Searches included a combination of Medical Subject Headings (MeSH) and keywords such as “crush syndrome”, “crush injury”, “rhabdomyolysis”, “renal failure”, “acute kidney injury”, “tissue necrosis”, “hyperbaric oxygen therapy”, “fluid resuscitation", and “trauma”. Boolean operators were used to optimize sensitivity and specificity. The search was limited to human studies published in English up to June 2025.

Eligibility Criteria

Studies were selected using the PICO framework [6] to ensure alignment with the review objectives. The population included patients experiencing crush injuries or crush syndrome, particularly in the context of trauma or disaster-related events. Interventions or exposures of interest involved pathophysiological processes linked to crush injuries or therapeutic strategies such as fluid resuscitation and HBOT. Comparisons were made against standard care, placebo or sham therapies, or the absence of specific interventions, where applicable. The outcomes assessed encompassed key domains such as systemic and localized pathophysiological responses (e.g., ischemia-reperfusion injury), anatomical consequences such as soft tissue necrosis, and renal outcomes including AKI and the requirement for dialysis. The review included randomized controlled trials, cohort studies, case series, and relevant narrative or systematic reviews that offered original clinical insights or evidence synthesis. Narrative reviews were incorporated selectively to enhance understanding of complex pathophysiological mechanisms where primary data were limited. Excluded materials included case reports, editorials, conference abstracts, animal studies, and studies lacking defined outcomes related to the review focus.

Study Selection

Two reviewers independently screened the titles and abstracts for relevance. Full-text articles of potentially eligible studies were then assessed for inclusion. Discrepancies were resolved through discussion or consultation with a third reviewer. A PRISMA flow diagram was used to document the selection process.

Data Extraction

Data were extracted independently by two reviewers using a structured extraction form. Key variables collected included author and year of publication, study design, population characteristics, type of injury, interventions/exposures, pathophysiological mechanisms discussed, anatomical regions affected, and renal outcomes.

Risk-of-Bias Assessment

Risk of bias for RCTs was assessed using the Cochrane Risk of Bias 2.0 tool [7]. For non-randomized studies, the Risk Of Bias In Non-randomized Studies of Interventions (ROBINS-I [8]) tool was applied. Narrative reviews were critically appraised using the SANRA (Scale for the Assessment of Narrative Review Articles) criteria [9] to ensure methodological soundness and relevance. All assessments were conducted independently by two reviewers, with consensus reached through discussion.

Data Synthesis

Due to the heterogeneity in study designs, outcome measures, and reporting styles, a narrative synthesis approach was adopted. Findings were thematically grouped into three main domains: (1) pathophysiological processes of crush syndrome, (2) anatomical and tissue-specific impacts, and (3) renal complications and therapeutic interventions. Comparative observations and interventional outcomes such as the efficacy of HBOT and fluid management protocols were highlighted where applicable.

Results

Study Selection Process

**Figure 1:**
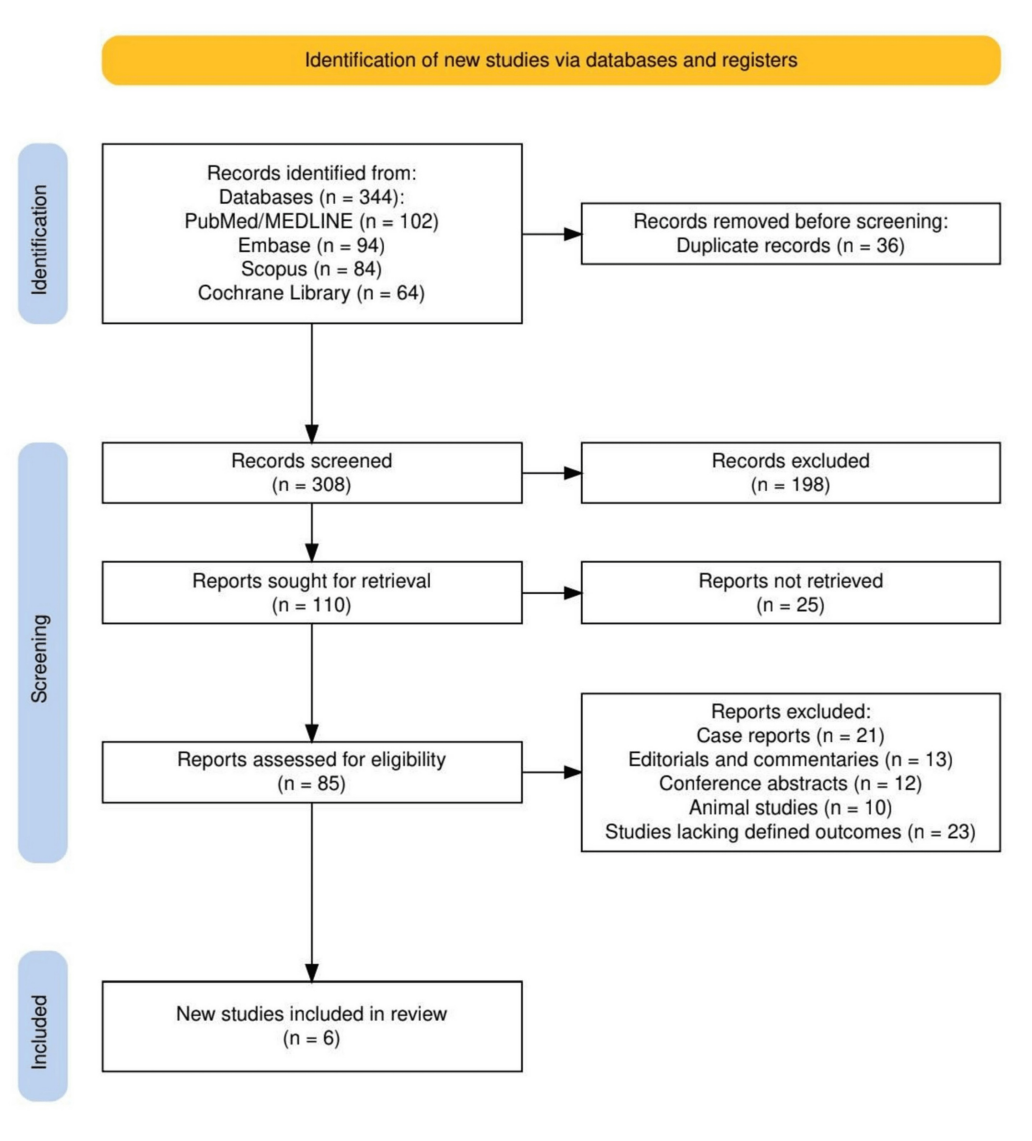
PRISMA flowchart representing the study selection process. PRISMA, Preferred Reporting Items for Systematic Reviews and Meta-Analyses

A total of 344 records were identified through database searches, including PubMed/MEDLINE (n = 102), Embase (n = 94), Scopus (n = 84), and the Cochrane Library (n = 64) (Figure 1). After removing 36 duplicates, 308 records were screened, of which 198 were excluded based on titles and abstracts. Following full-text assessment of 85 reports, 79 were excluded for reasons such as being case reports, editorials, conference abstracts, animal studies, or lacking defined outcomes. Ultimately, six studies were included in the final review.

Characteristics of the Selected Studies

The selected studies encompass a diverse population of trauma patients, particularly those affected by crush syndrome in disaster or high-impact injury settings (Table 1). The exposures evaluated range from pathophysiological events, such as rhabdomyolysis and ischemia-reperfusion injury, to therapeutic interventions, such as fluid resuscitation and HBOT. Most studies focused on systemic complications, especially renal outcomes such as AKI, and documented anatomical consequences including soft tissue necrosis. Table 1 provides a comparative overview of the studies' design, key findings, and clinical relevance in understanding crush-related trauma.

**Table 1:** Characteristics of the Selected Studies AKI, acute kidney injury; CK, creatine kinase; CS, crush syndrome; DIC, disseminated intravascular coagulation; HBOT, hyperbaric oxygen therapy; RRT, renal replacement therapy

Authors and Year	Population (P)	Exposure / Condition (I)	Comparator (C)	Outcomes (O)	Pathophysiological Findings	Anatomical Impact	Renal Outcomes
Usuda et al., 2023 [10]	Trauma patients, especially those in disaster/mass casualty settings	CS due to prolonged soft tissue compression	Not applicable	Mortality, shock, cardiac failure, sepsis, DIC, systemic inflammation	Ischemia-reperfusion injury, metabolic acidosis, hyperkalemia, systemic inflammatory response	Skeletal muscle injury from prolonged compression, soft tissue necrosis	Kidney dysfunction, need for early hemodialysis, risk of delayed AKI
Chavez et al., 2016 [11]	Patients with rhabdomyolysis (including trauma-induced)	Rhabdomyolysis and CS	Not applicable	Epidemiology, complications, and treatment outcomes	Muscle cell destruction → release of CK, myoglobin, potassium; systemic metabolic derangements	General skeletal muscle injury (not localized)	High risk of AKI due to myoglobinuria; fluid resuscitation emphasized; no conclusive data on bicarbonate/mannitol effectiveness
Akrivos et al., 2025 [12]	Patients with crush injuries, particularly in disaster and trauma settings	CS resulting from prolonged compression of soft tissues	Not applicable	Morbidity, mortality, need for dialysis, systemic complications	Ischemia–reperfusion injury, rhabdomyolysis, release of toxic metabolites (e.g., myoglobin, potassium), oxidative stress	Soft tissue and muscle necrosis, no detailed compartment-specific analysis	Risk of AKI due to rhabdomyolysis; early fluid therapy and electrolyte management emphasized; controversial role of mannitol and dialysis timing
Khan et al., 2025 [13]	Trauma patients with CS	CS due to traumatic rhabdomyolysis	Not applicable	Mortality, AKI, systemic toxicity, improved recovery with new therapies	Muscle necrosis releases myoglobin, potassium, lactic acid; ferroptosis, oxidative stress, macrophage-mediated injury	Musculoskeletal injury, fractures, skeletal muscle breakdown	AKI from iron overload and myoglobinuria; addressed with fluid therapy, antioxidants, RRT, and erythropoietin
Millar et al., 2022 [14]	Patients with open tibial fractures and soft tissue crush injuries	HBOT + standard trauma care	Standard trauma care alone	Tissue necrosis, infection, fracture union, complications, quality of life	Not directly analyzed; mechanism assumed to involve improved oxygenation and reduced ischemic damage	Severe lower limb soft tissue trauma; delayed fracture union evaluated	Not assessed
Kwee et al., 2024 [15]	Patients with crush-associated severe lower limb soft tissue injuries	HBOT + standard trauma care	Standard care or sham HBOT (varied by study)	Wound healing, infection rates, need for surgery, long-term complications	Not deeply discussed; implied benefit via enhanced oxygenation, tissue regeneration	Lower limb soft tissue trauma, open fractures, crush injuries	Not assessed

Risk-of-Bias Assessment

The risk-of-bias assessment revealed that most narrative reviews were rated as low-risk using the SANRA tool due to their comprehensive content and structured presentation, though inherent limitations exist due to their non-systematic nature (Table 2). One narrative review had a moderate risk due to limited methodological transparency. The included randomized controlled trial had some concerns using the RoB 2.0 tool, primarily related to lack of blinding. The systematic review assessed with AMSTAR 2 (A Measurement Tool to Assess Systematic Reviews 2) showed moderate risk, reflecting not only methodological strengths but also potential publication bias and heterogeneity in study design.

**Table 2:** Risk-of-Bias Assessment AMSTAR 2, A Measurement Tool to Assess Systematic Reviews 2; PRISMA, Preferred Reporting Items for Systematic Reviews and Meta-Analyses; RoB 2.0, Cochrane Risk of Bias Tool 2.0; SANRA, Scale for the Assessment of Narrative Review Articles

Study	Study Design	Risk-of-Bias Tool	Risk-of-Bias Rating	Justification
Usuda et al., 2023 [10]	Narrative review	SANRA	Low	Well-structured, referenced, and covers current knowledge comprehensively; however, inherent limitation due to narrative nature.
Chavez et al., 2016 [11]	Narrative review	SANRA	Moderate	Includes a broad scope and clinical relevance, but lacks structured methodology and source transparency.
Akrivos et al., 2025 [12]	Narrative review	SANRA	Low	Clear pathophysiology, therapeutic guidance, and critical discussion; aligns well with SANRA scoring criteria.
Khan et al., 2025 [13]	Narrative review	SANRA	Low	Recent, comprehensive review with novel pathophysiological insights and therapeutic updates; adheres to SANRA standards.
Millar et al., 2022 [14]	Randomized controlled trial	RoB 2.0	Some Concerns	Randomization and intervention are clear, but lack of blinding and limited reporting on adherence may introduce bias.
Kwee et al., 2024 [15]	Systematic review	AMSTAR 2	Moderate	Followed systematic methods and PRISMA principles, but mixed study designs and possible publication bias lower overall quality.

Discussion

Across the included studies, a consistent pattern emerged in the pathophysiological mechanisms underpinning crush syndrome, notably involving ischemia-reperfusion injury, rhabdomyolysis, and systemic inflammatory responses. Key metabolic derangements included hyperkalemia, lactic acidosis, and the release of myoglobin and creatine kinase from necrotic muscle tissues, contributing to multiorgan dysfunction. Usuda et al. [10] and Akrivos et al. [12] emphasized early hemodynamic compromise and the risk of AKI, with AKI noted as a major cause of mortality in such patients. Khan et al. [13] expanded on novel cellular pathways such as ferroptosis and oxidative stress-induced renal injury, highlighting the importance of timely antioxidant therapy. Regarding renal outcomes, both Chavez et al. [11] and Khan et al. [13] underscored myoglobinuria and iron overload as central to renal impairment, often necessitating dialysis. Fluid resuscitation remained a universal early intervention across studies, with the role of bicarbonate and mannitol yielding inconsistent support. Additionally, evidence from randomized controlled trials by Millar et al. [14] and Kwee et al. [15] demonstrated that HBOT significantly reduced tissue necrosis (29% vs. 53%, P = 0.01) and long-term complications (11.3% vs. 34.6%, P = 0.007), thereby offering promising adjunctive benefits, although renal outcomes were not directly evaluated in these HBOT-focused studies.

The findings of this review support existing understanding about the complex pathophysiology and clinical complications of crush syndrome in trauma patients. The transition from localized soft tissue injury to systemic effects such as ischemia-reperfusion injury and rhabdomyolysis is well recognized. Studies such as those by Akrivos et al. [12] and Chavez et al. [11] emphasize these mechanisms as central to clinical outcomes. New insights discussed in this review include the roles of ferroptosis and inflammatory macrophage activity in renal and systemic injury, adding depth to traditional models.

This review integrates evidence from various study types to provide a broad understanding of crush syndrome. It covers pathophysiological mechanisms, anatomical damage, and renal complications such as AKI [13,16]. Findings on the use of HBOT suggest a promising approach to improve tissue healing and reduce necrosis. At the same time, the clinical benefits of mannitol and bicarbonate remain uncertain, and further investigation is needed [17].

There are several limitations in the current literature. Few high-quality randomized controlled trials are available, especially those focusing on renal outcomes. Many included studies vary in design and outcome reporting, which may introduce bias. The exclusion of non-English articles and unpublished data may also limit the generalizability of the findings.

Clinically, this review highlights the importance of early fluid resuscitation, correction of electrolyte imbalances, and prompt identification of complications such as AKI. Adjunctive treatments such as HBOT appear beneficial in selected patients. Future research should focus on long-term renal outcomes, the role of antioxidant therapies, and the best practices for using hyperbaric oxygen. These steps are necessary to improve outcomes for patients with crush syndrome in trauma settings.

## Conclusions

In conclusion, this review consolidates current knowledge on the multifaceted nature of crush syndrome, emphasizing the interplay between pathophysiological derangements, anatomical damage, and renal complications, particularly AKI. It highlights the critical importance of early recognition and prompt, aggressive management, including fluid resuscitation and electrolyte correction, to mitigate systemic toxicity and improve patient outcomes. The emerging evidence supporting HBOT as an adjunctive intervention marks a promising shift toward reducing tissue necrosis and long-term disability, though standardized protocols remain to be established. Despite limitations in high-quality trials, this synthesis underscores the need for a coordinated, evidence-informed approach to crush injury management, while also identifying key research priorities such as optimizing renal protection strategies, clarifying the role of controversial agents such as mannitol, and advancing novel therapies targeting oxidative stress.
